# Minimum quality threshold in pre-clinical sepsis studies (MQTiPSS): an international expert consensus initiative for improvement of animal modeling in sepsis

**DOI:** 10.1186/s40635-018-0189-y

**Published:** 2018-08-14

**Authors:** Marcin F. Osuchowski, Alfred Ayala, Soheyl Bahrami, Michael Bauer, Mihaly Boros, Jean-Marc Cavaillon, Irshad H. Chaudry, Craig M. Coopersmith, Clifford Deutschman, Susanne Drechsler, Philip Efron, Claes Frostell, Gerhard Fritsch, Waldemar Gozdzik, Judith Hellman, Markus Huber-Lang, Shigeaki Inoue, Sylvia Knapp, Andrey V. Kozlov, Claude Libert, John C. Marshall, Lyle L. Moldawer, Peter Radermacher, Heinz Redl, Daniel G. Remick, Mervyn Singer, Christoph Thiemermann, Ping Wang, Willem Joost Wiersinga, Xianzhong Xiao, Basilia Zingarelli

**Affiliations:** 1 Ludwig Boltzmann Institute for Experimental and Clinical Traumatology in the AUVA Research Center, Donaueschingenstrasse 13, A-1200 Vienna, Austria; 2 Rhode Island Hospital & Alpert School of Medicine at Brown University, Providence, RI USA; 3 Jena University Hospital, Jena, Germany; 4 Institute of Surgical Research, University of Szeged, Szeged, Hungary; 5 Institut Pasteur, Paris, France; 6 University of Alabama at Birmingham School of Medicine, Birmingham, AL USA; 7 Emory University School of Medicine, Atlanta, GA USA; 8 Feinstein Institute for Medical Research, Northwell Health, Manhasset, NY USA; 9 University of Florida College of Medicine, Gainesville, FL USA; 10 Division of Anaesthesia and Intensive Care, Karolinska Institutet, Danderyd Hospital, Stockholm, Sweden; 11 AUVA Traumacenter, Vienna, Austria; 12 Paracelsus Medical University, Salzburg, Austria; 13 Wroclaw Medical University, Wroclaw, Poland; 14 School of Medicine, University of California, San Francisco, San Francisco, CA USA; 15 Institute of Clinical and Experimental Trauma-Immunology, University Hospital of Ulm, Ulm, Germany; 16 Kobe University Graduate School of Medicine, Kobe, Japan; 17 Department of Medicine 1, Medical University Vienna, Vienna, Austria; 18 Center for Inflammation Research, VIB, Ghent, Belgium; 19 University Ghent, Ghent, Belgium; 20 Keenan Research Centre for Biomedical Science, St. Michael’s Hospital, University of Toronto, Toronto, Canada; 21 Institute of Anaesthesiological Pathophysiology and Process Development, University Hospital of Ulm, Ulm, Germany; 22 Boston University School of Medicine, Boston, MA USA; 23 Bloomsbury Institute of Intensive Care Medicine, University College London, London, UK; 24 The William Harvey Research Institute, Barts and London School of Medicine & Dentistry, Queen Mary University of London, London, UK; 25 Feinstein Institute for Medical Research, Manhasset, NY USA; 26 Division of Infectious Diseases, and Center for Experimental and Molecular Medicine, the Academic Medical Center, University of Amsterdam, Amsterdam, The Netherlands; 27 Xiangya School of Medicine, Central South University, Chagnsha, Hunan China; 28 Division of Critical Care Medicine, Cincinnati Children’s Hospital Medical Center, College of Medicine, University of Cincinnati, Cincinnati, OH USA

**Keywords:** Guidelines, Experiment, Study design, Humane modeling, Infection types, Organ dysfunction, Fluid resuscitation, Antimicrobial therapy

## Abstract

**Background:**

Pre-clinical animal studies precede the majority of clinical trials. While the clinical definitions of sepsis and recommended treatments are regularly updated, a systematic review of pre-clinical models of sepsis has not been done and clear modeling guidelines are lacking.

**Objective:**

To address this deficit, a Wiggers-Bernard Conference on pre-clinical sepsis modeling was held in Vienna in May 2017. The goal of the conference was to identify limitations of pre-clinical sepsis models and to propose a set of guidelines, defined as the “*Minimum Quality Threshold in Pre-Clinical Sepsis Studies*” (MQTiPSS), to enhance translational value of these models.

**Methods:**

A total of 31 experts from 13 countries participated and were divided into 6 thematic working groups (WG): (1) study design, (2) humane modeling, (3) infection types, (4) organ failure/dysfunction, (5) fluid resuscitation, and (6) antimicrobial therapy endpoints. As basis for the MQTiPSS discussions, the participants conducted a literature review of the 260 most highly cited scientific articles on sepsis models (2002–2013).

**Results:**

Overall, the participants reached consensus on 29 points; 20 at “recommendation” (R) and 9 at “consideration” (C) strength. This executive summary provides a synopsis of the MQTiPSS consensus (Tables 1, 2, and 3). Detailed commentaries to all Rs and Cs are simultaneously published in three separate full-length papers.

**Conclusions:**

We believe that these recommendations and considerations will serve to bring a level of standardization to pre-clinical models of sepsis and ultimately improve translation of pre-clinical findings. These guideline points are proposed as “best practices” for animal models of sepsis that should be implemented. In order to encourage its wide dissemination, this article is freely accessible in Shock, Infection and Intensive Care Medicine Experimental.

“This modeling thing, it's pretty easy, but actually it's also really tough.” Cara Delevingne

## Review

### The necessity

With the ultimate goal to reduce mortality/morbidity in patients, animal modeling of diseases has been limited by poor translation [1, 2]. This is often fueled by the low fidelity of available model systems [3, 4], their inappropriate study designs [2], and selective use of animal data [5, 6]. When compared to other inflammatory states (e.g., arthritis, atherosclerosis), the complexity of sepsis has hampered the development of high-fidelity models. However, this challenge can be aptly embraced by building on recent advances in the understanding of sepsis pathophysiology and avoiding past errors. Any promising sepsis model must be (a) specifically tailored to the posited hypothesis, (b) “*reverse translated*” to its clinical counterpart [7, 8], and (c) adjusted as new pathophysiological evidence emerges. This is echoed by the US Food and Drug Administration (FDA) in their 2010 Guidance for Industry and FDA Staff: “*FDA believes that the animal…(model)…should provide a test system that offers a best attempt at simulating the clinical setting*” (General Considerations for Animal Studies for Cardiovascular Devices; https://www.fda.gov/MedicalDevices/ucm220760.htm).

Unfortunately, while the clinical definition of sepsis is currently in its third iteration [9] and the Surviving Sepsis Campaign (SSC) Guidelines for patient management have been updated three times [10], pre-clinical sepsis research has not been subjected to any organized attempt at introducing best practices, management guidelines, and standardization [11]. This creates a large quality gap and confusion with conflicting data reflecting huge variations in, for example, insult severity, fluid resuscitation, and study duration. Effective animal modeling and reporting guidelines have recently been proposed for other specific diseases such as pulmonary fibrosis [12], stroke [13, 14], heart failure [15], and malaria [16] making the void in the field of pre-clinical sepsis even more apparent. It is essential that animal models of sepsis continue to evolve. Lack of sufficient standardization of pre-clinical models will continue to limit the utility of sepsis animal research as a useful platform for advancing clinical outcomes and care in sepsis [17, 18] and will reduce the opportunities to identify and test new therapies.

### The action

To address this perceived deficit, the Ludwig Boltzmann Institute of Experimental and Clinical Traumatology in the AUVA Research Center organized in May 2017 in Vienna a Wiggers-Bernard Conference on “*Pre-clinical Modeling in Sepsis: Exchanging Opinions and Forming Recommendations*.” The key goal was to create publishable material that characterizes elements that should be included in pre-clinical sepsis studies and defined by the so called “*Minimum Quality Threshold in Pre-Clinical Sepsis Studies*” (MQTiPSS) descriptor. The Wiggers-Bernard Conference participants identified and addressed several broad, critically important concepts in animal sepsis modeling. A total of 31 experts from 13 countries participated in the initiative (including five members of the Sepsis-3 definitions task force) and were divided into six thematic Working Groups: (1) study design, (2) humane endpoints, (3) infection types, (4) organ failure/dysfunction, (5) critical fluid resuscitation, and (6) antimicrobial therapy.

The initiative consisted of three phases: (a) preparatory (prior to the meeting; approximately 3 months), during which participants performed a systematic review of the 260 top-cited (over 29,000 citations in aggregate) 2003–2012 pre-clinical publications (using ISI Web of Knowledge database; query: “sepsis model;” 374 individual experiments analyzed) and identified the key modeling topics to be discussed; (b) discussion during which the participants spent 2 days at the Wiggers-Bernard Conference examining pre-clinical sepsis models and ultimately voted to reach consensus on the proposed points (either at the “recommendation” or “consideration” strength); and (c) post-meeting refinement of the accepted points and finalization of the arguments to be included in the final publications (using a modified Delphi method; approximately 3 months). Following the format used by the Sepsis-3 task force [8], at least two thirds (over 65%) of the votes were required for approval of a proposed point.

### The proposed outcome

First, a definition for an animal model of sepsis was formulated and (unanimously) approved: “*An experimental animal (mammal) model of sepsis should be defined as life-threatening organ dysfunction caused by a dysregulated host response to an infection.*” Second, Wiggers-Bernard Conference participants reached consensus on 29 points; 20 at “recommendation” strength and 9 at “consideration” strength (listed in Tables 1, 2, and 3). All consensus points were reached either unanimously or with no more than two abstentions per point (point 8). The “recommendation” strength indicates virtually unanimous agreement among the 31 participants, regarding both the content and the need for rapid implementation. Issues that require additional discussion before final recommendations could be made were classified as considerations.

**Table 1:** Combined recommendations and considerations from the working groups (WG) 1 and 2

Study design (WG-1)	1. Survival follow-up should reasonably reflect the clinical time course of the sepsis model	R
	2. Therapeutic interventions should be initiated after the septic insult replicating clinical care	
	3. We recommend that the treatment be randomized and blinded when feasible	
	4. Provide as much information as possible (e.g., ARRIVE guidelines) on the model and methodology, to enable replication	
	a. Consider replication of the findings in models that include co-morbidity and/or other biological variables (i.e., age, gender, diabetes, cancer, immuno-suppression, genetic background, and others)	C
	b. In addition to rodents (mice and rats), consider modeling sepsis also in other (mammal) species	
	c. Consider need for source control	
Humane modeling (WG-2)	5. The development and validation of standardized criteria to monitor the well-being of septic animals is recommended	R
	6. The development and validation of standardized criteria for euthanasia of septic animals is recommended (exceptions possible)	
	7. Analgesics recommended for surgical sepsis consistent with ethical considerations	
	d. Consider analgesics for nonsurgical sepsis	C

*R* recommendation strength, *C* consideration strength

**Table 2:** Combined recommendations and considerations from the working groups (WG) 3 and 4

Infection types (WG-3)	8. We recommend that challenge with LPS is not an appropriate model for replicating human sepsis	R
	9. We recommend that microorganisms used in animal models preferentially replicate those commonly found in human sepsis	
	e. Consider modeling sepsis syndromes that are initiated at sites other than the peritoneal cavity (e.g., lung, urinary tract, brain)	C
Organ Failure/ Dysfunction (WG-4)	10. Organ/system dysfunction is defined as life-threatening deviation from normal for that organ/system based on objective evidence	R
	11. Not all activities in an individual organ/system need to be abnormal for organ dysfunction to be present	
	12. To define objective evidence of the severity of organ/system dysfunction, a scoring system should be developed, validated and used, or use an existing scoring system	
	13. Not all experiments must measure all parameters of organ dysfunction but animal models should be fully exploited	
	f. Avoid hypoglycemia	C

*R* recommendation strength, *C* consideration strength

**Table 3:** Combined recommendations and considerations from the working groups (WG) 5 and 6

Fluid Resuscitation (WG-5)	14. Fluid resuscitation is essential unless part of the study	R
	15. Administer fluid resuscitation based on the specific requirements of the model	
	16. Consider the specific sepsis model for the timing of the start and continuation for fluid resuscitation	
	17. Resuscitation is recommended by the application of iso-osmolar crystalloid solutions	
	g. Consider using pre-defined endpoints for fluid resuscitation as deemed necessary	C
	h. Avoid fluid overload	
Antimicrobial Therapy (WG-6)	18. Antimicrobials are recommended for pre-clinical studies assessing potential human therapeutics	R
	19. Antimicrobials should be chosen based on the model and likely/known pathogen	
	20. Administration of antimicrobials should mimic clinical practice	
	i. Antimicrobials should be initiated after sepsis is established	C

*R* recommendation strength, *C* consideration strength

The current executive summary briefly describes the Wiggers-Bernard Conference initiative and presents the compiled consensus points. The details of the recommendations/considerations are published in three separate papers [19–21] appearing in the December issue of Shock. Tables 1, 2, and 3 summarize the main MQTiPSS consensus points published in those articles: part I—Table 1 content [19], part II—Table 2 [20], and part III—Table 3 [21]. Each publication is built on two (related) working group themes and includes a narrative clarifying caveats and intricacies related to the accepted consensus points.

### The future

The presented consensus has not received formal endorsement from professional bodies. Writing an initial consensus was a strategic decision given that an expert opinion report has a shorter publication turnaround and our intention was to rapidly introduce the MQTiPSS concept. The Wiggers-Bernard Conference was conceived not as a one-time event but rather as an initial “call-to-arms,” an invitation to interested parties to provide further refinement and expansion of the proposed points. The on-going expansion initiatives include formation of a task force (under the auspices of the Shock Society; June 2017) for creation of robust, defined parameters to score sepsis models for clinical relevance. Another iteration of the Wiggers-Bernard Conference on animal sepsis models is planned for October 2019 at the joint conference of the European Shock Society and International Federation of Shock Societies in Crete, Greece.

## Conclusions

In summary, we believe that the proposed guidelines represent the first concrete steps toward creation of a realistic framework for standardization of animal models of sepsis (i.e., MQTiPSS). Such a framework, once widely employed, will improve the quality of pre-clinical investigation and arm clinicians with better tools for combating sepsis in patients.

## Abbreviations

C: Consideration
FDA: the US Food and Drug Administration
MQTiPSS: Minimum Quality Threshold in Pre-Clinical Sepsis Studies
R: Recommendation
SSC: the Surviving Sepsis Campaign
